# A human rights approach to mental health and people with disabilities

**DOI:** 10.2471/BLT.18.030818

**Published:** 2018-08-01

## Abstract

Dainius Puras has devoted the last 30 years to protecting the rights of children with mental disabilities and of other vulnerable groups. He talks to Fiona Fleck.

Q: How did you become interested in mental health and the rights of people with mental health disabilities? 

A: Many of my fellow medical students were interested in surgery and other classically biomedical subjects, but already I felt that medicine was not just about repairing parts of the human body. When I graduated, the university needed a teacher for child and adolescent psychiatry and invited me to be an assistant professor. I did my PhD on what was known as “mental retardation” or “oligophrenia”. My first patients were children with developmental disabilities, such as autism and Down syndrome. At that time Lithuania was part of the Soviet Union that wanted to showcase the achievements of the Communist state, as if all problems had been solved by the system. The state claimed it was looking after all its people’s needs and that human rights were protected, but the reality was very different. 

Q: How was it different? 

A: Parents struggled to get care for their children with mental disabilities because the state pretended they did not exist. As a doctor, you were trained and expected to advise parents to abandon such children in horrible residential institutions for the rest of their lives. If parents refused, they were deprived of services for such children in the community. They told me how they had to confine these children to their homes, only taking them out for walks at night. Even my teachers, otherwise good teachers and clinicians, shared this value system that held that children and adults with serious conditions have no future prospects and should be abandoned. One of my first patients in the women’s psychiatric unit was diagnosed with schizophrenia. My teacher invited her husband to talk to us and strongly recommended him to divorce her. This seemed wrong to me. Then when I started to express my concerns, I was dismissed by colleagues as “always talking about human rights” which was considered inappropriate in that highly medicalized system, that insisted on aggressive treatment and cure.

Q: The Soviet Union was often criticized for human rights violations. How easy was it to take a human rights approach to health? 

A: It was the late 1980s. The Soviet leader Mikhail Gorbachev had launched the policies of *glasnost* (openness) and *perestroika* (reconstruction), allowing limited economic and civil freedoms. This provided a window of opportunity to promote the rights of children with mental disabilities. Then after 1991, when the Soviet Union collapsed, there were opportunities in the new democracies, including my own country.

“Your children’s future is in your hands, now that we have democracy”

Q: How did you start? 

A: I knew from good practice in other countries that an initiative must come from affected citizens. I’d wanted to do this since the early 1980s, but in the Soviet Union it was illegal for an interest group to openly address a critical issue or establish an NGO, so I waited. Then when we saw the first glimpses of democracy in 1989, I took the opportunity and placed an advertisement in a newspaper inviting the parents of children with mental disabilities to meet. Many people came, most of them the mothers of such children. It was the first time they’d ever met each other. I asked them whether they wanted to change their children’s lives. “Your children’s future is in your hands, now that we have democracy”, I said. 

Q: What was their response?

A: They asked me to be their chairperson but I declined, saying I was happy to advise them to help empower them. They were disappointed but soon realised what I meant. They understood their children’s needs better than many doctors and they knew how to put pressure on the government to provide appropriate services. The parents formed a group called *Viltis (“*hope” in Lithuanian). At first “hope” referred to their dream that – now we were allowed to travel abroad – they could take their children to a clinic in London or Paris to be cured. I explained that it was not about finding a medical cure, and so “hope” took on a new meaning: the hope that their children could live with dignity – not stigmatized and hidden, neglected by society – and that when parents become older, their children could live as independently as possible and not be institutionalized. 

Q: How did you bring about change in the way the state supports these children in Lithuania? 

A: I initiated a child development service at the university hospital that looked at how to provide services for these children and families in a new way. We introduced new methods of managing developmental disabilities and mental health problems in children and adolescents. We implemented a system for child mental health care and rehabilitation for childhood disabilities. We developed innovative services for children with mental and developmental problems and disorders across the country. This is still a work in progress.

Q: You became the president of the newly established Lithuanian Psychiatric Association in 1990. How has this society contributed to modernizing mental health care provision? Are you satisfied with the results? 

A: I hoped that within 10 years, Lithuanian psychiatry would have embraced the ethical principles of other developed countries. This has not happened so far. The priority for Lithuanian psychiatry is still to provide biomedical treatment, which is important as long as the human rights of people with mental health conditions are respected, and this is not always the case. Lithuania, with a population of 3 million people, has 6000 adults and 3000 children in closed institutions. Lithuania is not an exception, reliance on such an outdated and ineffective system is still in place in many neighbouring countries. Professional groups should be the first to pressure the government to stop depriving people of their liberty. When political reforms took place in eastern Europe in the 1990s, many people were not ready to exercise new freedoms.

Q: Why? 

A: In the era of Soviet communism, the state took care of everyone, providing access to health care and protecting social and economic rights. There were no homeless people. Everyone had a job. But this protection was all at the expense of civil and political rights. To have good public health, all rights including civil and political rights must be protected. The Soviet system protected people in selective way, it didn’t empower them. The state sort of said “We will take care of you, but critical views and private initiatives are not allowed”. With the advent of the market economy and open society, people had to take much more responsibility for their own lives. After living in a benevolent prison, many people lacked the effective coping skills needed to survive in an open society. That is when the epidemic of destructive and self-destructive behaviours started. Many people felt they had lost out and regressed, resulting in high rates of alcohol abuse, violence and suicide. Men showed much less resilience than women, and since then men’s life expectancy is much lower than that of women. 

Q: How are countries of central and eastern Europe addressing this toll of mental health problems? 

A: The mental health system is heavily dependent on institutional care, medications and psychiatric hospitals, not only in this region but all over the world. This is something I highlight in my thematic reports and country missions, as UN Special rapporteur. A woman is unhappy because she suffers from domestic violence. She cannot sleep, she’s told to go to a psychiatrist who says “You are depressed, here are some pills”. We prescribe psychotropic medications to solve social problems. Power imbalances in our societies and the resulting injustices and distortions are interpreted by doctors and textbooks as “chemical imbalances” in the brain. This is a global problem that I raised in my report on mental health in 2017, which was critical overview of psychiatry globally. 

Q: Which human rights and health issues do you raise with countries as the Special Rapporteur on the right to health? 

A: When I visit countries to report on the right to health, I often visit closed psychiatric institutions and make recommendations on how a country can move to a modern system based on evidence and human rights. That means that patients should have a say in the treatment they receive, and that innovative approaches are used to avoid coercion, which is harmful for therapeutic relationships. Often less developed countries say “We’re not rich, we can’t develop rights-based services”, but they should know that long-term investment in closed institutions breeds hopelessness and exclusion, and may be even more expensive. The conditions in closed institutions are often terrible, overcrowded and unsanitary and it’s expensive to improve them. So why lock people up? Why violate people’s rights? One of my priorities is to convince politicians and leaders in psychiatry that they need to change the system. 

Q: Human rights abuses of people with mental health problems are well documented. People do not always associate psychiatry with human rights, how do you address this in your work? 

A: I have been involved in all kinds of reforms in Lithuania, including health, education and social reforms, and later in policy reforms in several other countries including Bulgaria, Georgia and Ukraine. I also served several years as a WHO mental health national counterpart . People would often ask me “You are a medical doctor, why are you so interested in human rights?”, reflecting the misconception that there is no connection between medicine and human rights and the neglect of the basic principle of medicine: do no harm. Undermining human rights and the principle of informed consent has led to violations of human rights in the history of medicine. The global community needs to learn from this experience. As an independent expert appointed by the UN, I am glad to have the opportunity to convey these important messages globally, when I present my reports. 

Q: Do all the people locked in such institutions have recognizable psychiatric conditions or are they confined for non-medical reasons? 

A: People are confined for various reasons. This may be for mental health conditions, tuberculosis, leprosy, HIV, or other communicable diseases. This may be for drug and alcohol use, behavioural problems in children and adolescents, and so on. Unfortunately the confinement is used too often to manage public health issues, when in fact modern evidence-based policies are needed. Policymakers should avoid policies based on confinement. There are many effective alternatives to those outdated practices and policies. 

